# Comparison of subclinical dermatophyte infection in short- and long-haired cats

**DOI:** 10.14202/vetworld.2020.2798-2805

**Published:** 2020-12-29

**Authors:** Panpicha Sattasathuchana, Chunyaput Bumrungpun, Naris Thengchaisri

**Affiliations:** 1 Department of Companion Animal Clinical Sciences, Kasetsart University, Bangkok 10900, Thailand; 2 The Veterinary Diagnosis Laboratories, Faculty of Veterinary Medicine, Kasetsart University, Bangkok, 10900, Thailand

**Keywords:** cats, dermatophyte, hematology, mycosis, Wood’s lamp

## Abstract

**Background and Aim:**

Long-haired cats may have an increased risk of dermatophytosis due to insufficient grooming and their thick hair coat trapping fungal spores. The prevalence of subclinical dermatophytosis in long-haired cats was evaluated using fungal culture and Wood’s lamp test. Hematology and blood chemistry results were compared between cats negative and positive for dermatophytosis.

**Materials and Methods:**

A total of 127 cats (median age, 3 years [range, 10 months-10 years]) without feline leukemia virus or feline immunodeficiency virus infection were classified into short-haired (n=64) and long-haired (n=63) groups. Hair samples were cultured on a fungal culture medium (dermatophyte test medium, enhanced sporulation agar, and Sabouraud agar).

**Results:**

The prevalence of dermatophytosis in short-haired and long-haired cats was 6.25% (95% confidence interval [CI], 2.15-12.28) and 34.92% (95% CI, 22.94-46.90), respectively. The odds of long-haired cats having dermatophytosis were 8.05 (95% CI, 2.44-33.97) times greater than that in short-haired cats. The number of positive dermatophytosis found in domestic short-haired cats (2/50, 4.0%) was significantly lower than that in Persian cats (17/47, 36.17%; p<0.001) and long-haired mixed breed cats (3/7, 42.86%; p=0.011). The overall sensitivity and specificity of the Wood’s lamp test for diagnosing *Microsporum canis* infection were 37.5% (95% CI, 21.2-57.3%) and 96.1% (95% CI, 90.4-98.5%), respectively. Cats with dermatophytosis had significantly lower hematocrit and serum albumin levels than cats without dermatophytosis.

**Conclusion:**

Subclinical dermatophytosis was more common in long-haired cats; therefore, dermatophyte examinations should be performed routinely.

## Introduction

Dermatophytosis is a common and highly contagious zoonotic disease seen in cats [1,2]. *Microsporum canis* is the most commonly isolated pathogen in feline dermatophytosis [2]. *Microsporum persicolor*, *Microsporum gypseum*, *Trichophyton verrucosum*, *Trichophyton mentagrophytes*, and *Epidermophyton* species have been isolated in a few cats [2-6]. Diagnostic methods, such as skin cytology, biopsy, polymerase chain reaction, and fungal cultures, have been used to detect dermatophyte infection [1,3,6-13]. Although Wood’s lamp is the fastest dermatophytosis test, it has a low sensitivity [3]. Fungal culture provides highly specific test results for detecting dermatophytosis, but it is time-consuming [1,12]. Numerous testing media have been used to isolate and identify dermatophyte species [12]. Dermatophyte test medium (DTM) and enhanced sporulation agar (ESA) are selective dermatophyte test media containing dermatophyte growth enhancers and dye indicators [1,14,15], and Sabouraud dextrose agar (SDA) is a non-specific, classic universal agar that is used to grow saprophytes [1]. Dermatophytes quickly grow on SDA [12], and a combination of DTM, ESA, and SDA increases the ability to detect dermatophytosis and other fungal diseases.

Dermatophytes are not normal fungal flora of cats [16]. Isolation of these fungal species in healthy animals indicates subclinical infection and asymptomatic transient carriage [2,17,18]. Important factors related to dermatophyte infection include age, lifestyle, living situation (indoor vs. outdoor), and climate of its living area (outdoor cats living in warmer climates are more susceptible to dermatophytosis) [1]. Cats of all ages can contract the disease, but kittens are commonly affected [1,5]. It is still unclear whether cats that are infected with feline leukemia virus (FeLV) or feline immunodeficiency virus (FIV) are more susceptible to dermatophyte infection [10,17,18]. Although long-haired cats have a long and thick coat in which fungal spores may become trapped, the relationship between coat length and infection with dermatophytes remains unclear.

The present study aimed to: (1) Identify and compare the prevalence of dermatophytosis in short- and long-haired cats without FeLV and FIV ­infection; (2) determine the association between dermatophytosis and hair length; (3) evaluate the sensitivity, specificity, positive predictive value, and negative predictive value of the Wood’s lamp test for dermatophytosis detection; and (4) compare hematology and blood chemistry parameters of cats with and without dermatophytosis.

## Materials and Methods

### Ethical approval and informed consent

The sample collection protocols were reviewed and approved by the Animal Care and Use Committee at Kasetsart University (ACKU60-VET-032). The informed owner consent forms were signed before samples were collected.

### Study period and location

The samples were collected from cats visiting Kasetsart University Veterinary Teaching Hospital, Bangkok, Thailand, for routine health check between July 2017 to June 2018.

### Animals

A total of 127 privately owned indoor cats were enrolled in the hematology and dermatophytosis survey. The age, sex, breed, and hair length of the cats were recorded. The cats were divided into two groups: Short-haired and long-haired cats. Cats with a hair shaft length <3 cm were classified as the short-haired cat group and the other cats were classified as the long-haired cat group. A general physical examination was performed on all cats. None of the cats showed dermatological signs. Approximately 3 mL of blood was collected through the jugular vein and transferred to ethylenediaminetetraacetic acid-coated tubes and plain glass tubes for complete blood count and blood chemistry testing, respectively. Complete blood count was performed using an automated hematology analyzer (Abbott CELL-DYN 3700 Hematology Analyzer, Abbott, Germany) to measure hematocrit, white blood cell count, neutrophil count, lymphocyte counts, monocyte count, and eosinophil count. Blood chemistry was performed using a laboratory chemical analyzer (ILab650 Automatic Biochemistry analyzer, Instrumental Laboratory, USA) to measure blood urea nitrogen, creatinine, alanine aminotransferase, aspartate aminotransferase, alkaline phosphatase, total protein, albumin, and globulin. Cats with confirmed positive results for FeLV or FIV using rapid immunochromatographic tests (FASTest FeLV-FIV, MEGACOR Diagnostik GmbH, Austria) were excluded from the study.

### Wood’s lamp skin examination

**Figure-1:**
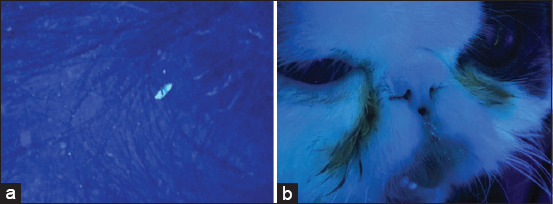
The images of the positive Wood’s lamp test of short-haired and long-haired cats. (a) A bright green fluorescent spot was detected on the base of a hair shaft over the truncal area in a short-haired cat with positive to *Microsporum canis*. (b) Green flurescencing facial hairs were identified in a long-haired cat with positive to *M. canis*.

All cats were tested for *M. canis* using Wood’s lamp test in a dark room. A glowing hair shaft with a bright apple green to yellowish color was considered a positive result (Figure-1). Both fluorescent colors of scale and non-fluorescent color were considered a negative test result.

### Fungal culture

**Figure-2:**
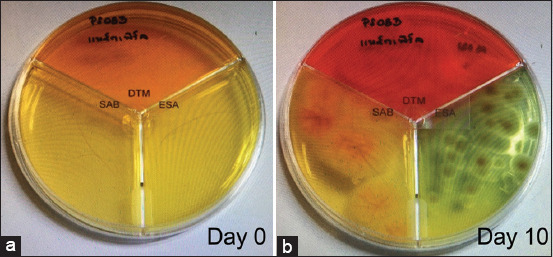
The MYCODERMOASSAY Trio was used for rapid detection of dermatophyte. (a) Hair samples (day 0) from each cat were cultured on the MYCODERMOASSAY Trio. (b) Early diagnosis can be made by detecting the color changes on the fungal plate (day 10). The characteristic of dermatophyte colony can be found in Sabouraud (SAB) agar. Color changes from orange to red can be detected in the dermatophyte test medium for positive dermatophyte colony. Color changes from yellow to green can be detected in the enhanced sporulation agar (ESA) for positive dermatophyte colony. Microscopic differentiation of dermatophytes can be visualized using colony from the ESA.

**Figure-3:**
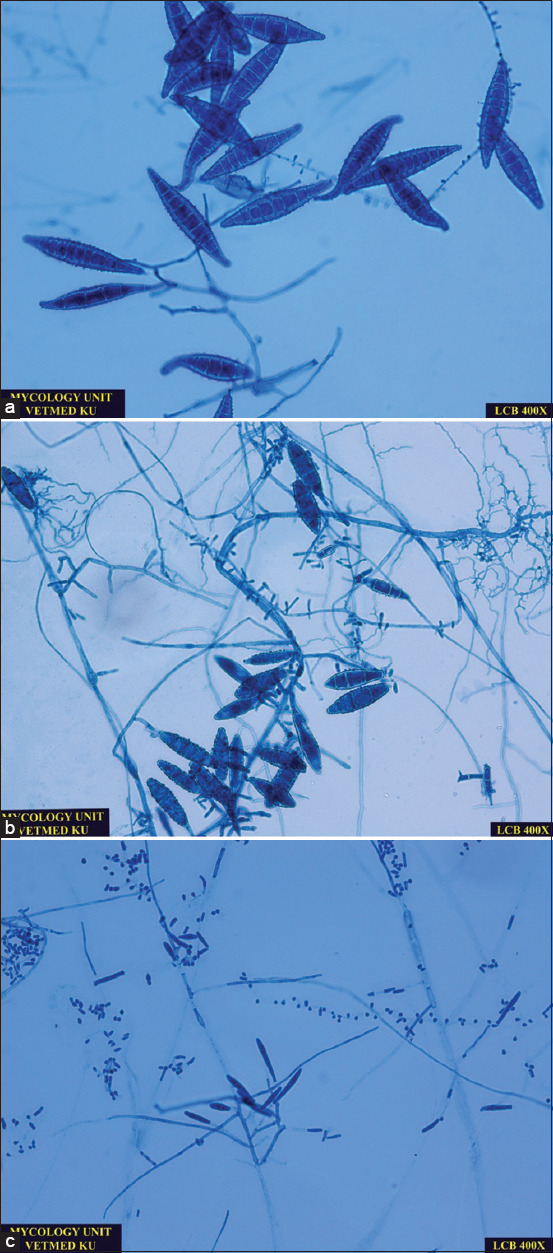
Microscopic images of common dermatophyters identified in cats using the lactophenol cotton blue staining. (a) *Microsporum canis* is confirmed by a presence of spindle-shaped, rough, thick-walled, macroconidia, and few round-shaped microconidia (400×). (b) *M. gypseum* is confirmed by a presence of boat-shaped, rough, thin-walled macroconidia and few club-shaped microconidia (400×). C: *Trichophyton mentagrophytes* is confirmed by a presence of cigar-shaped, smooth, thin-walled macroconidia and abundant grape-like clusters of microconidia (400×).

Hair and scale samples were collected for fungal culture using Mackenzie’s toothbrush technique in which a sterile toothbrush was used to brush the hair coat for 20 strokes or at least 2 min [1]. Hair shafts and scales were transferred to a combi culture agar plate containing three separate media (DTM, ESA, and SDA; MYKODERMOASSAY Trio, MEGACOR Diagnostik GmbH, Austria). Agar plates were then incubated at room temperature (25°C-30°C). The DTM and ESA culture agars were closely monitored for color changes. Dermatophyte colonies were identified by a change in the orange color of the DTM agar and the yellow color of the ESA agar to red and green-blue, respectively (Figure-2). Colony growth on the DTM, ESA, and SDA culture agars was monitored for 21 days [12]. Microscopic examination of wooly, fluffy colonies was performed on all positive agars to identify the fungal hyphae and spores, as shown in Figure-3. All fungal species were recorded. After 21 days, any non-growing colonies and media showing no color change were discarded and reported as negative for fungi.

### Statistical analysis

Statistical analyses were performed using commercially available statistical software packages (JMP Pro 10, SAS Institute, Cary, North Carolina, USA; GraphPad Prism version 6.0, GraphPad Software, Inc., La Jolla, California, USA; STATA v 12, StataCorp, College Station, Texas, USA). The significance level was set at p<0.05. Shapiro–Wilk W test was used to assess the normality of the data. Student’s t-test was performed to compare the age and body weight of the cats. Comparison of the categorical variables, including sex, was performed using the Pearson χ^2^ test.

Positive test results from the dermatophyte cultures were used to calculate the prevalence and the 95% confidence interval (CI) of dermatophytosis in the long- and short-haired cats. The association between hair length and each type of fungal species was determined using Fisher’s exact test, and the odds ratio of dermatophytosis in long-haired cats also was calculated. The sensitivity, specificity, positive predictive value, negative predictive value, and CI of the Wood’s lamp for diagnosing *M. canis* infection also were determined. Hematology and blood chemistry parameters were compared between cats with dermatophytosis and cats without dermatophyte infection. Student’s t-test and Wilcoxon rank-sum test were selected to apply on parametric data and non-parametric data, respectively. The significance level was set at p<0.05.

## Results

The demographic characteristics of the cats, including age (p=0.8847) and sex (p=0.1106), were not significantly different between the short- and long-haired groups (Table-1). However, the short-haired cats weighed more than the long-haired cats (p=0.0131).

**Table-1:** Demographic distribution of 127 cats enrolled in the present study.

Characteristic	Short-haired cats	Long-haired cats	p-value
n	64	63	-
Age (years)			0.8847
	3.2 (0.9-13)	3 (0.8-11)	
Sex, % (n.)			0.1106
Male	60.9% (39)	54.0% (34)	
Female	39.1% (25)	46.0% (29)	
Body weight, kg			0.0131
Median (range)	4.4 (2.4-9.5)	3.8 (2.1-10.2)	
Breed, % (n.)			
Domestic short haired	78.1% (50)	-	-
Scottish fold	7.8% (5)	-	-
American or British short hair	14.1% (9)	-	-
Persian	-	74.6% (47)	-
Maine coon	-	11.1% (7)	-
Mixed breed (hair shaft length >3 cm)	-	14.3% (9)	-

Dermatophyte infection was identified in 26 cats (Table-2), with *Microsporum* and *Trichophyton* species isolated in 24 and 2 cats, respectively. Other fungi were also identified, including *Absidia* species (n=8), *Aspergillus* species (n=43), *Bipolaris* species (n=5), *Chaetonium* species (n=2), *Cladophialophora* species (n=2), *Cladosporium* species (n=12), *Curvularia* species (n=5), *Nigrospora* species (n=2), *Paecilomyces* species (n=3), *Penicillium* species (n=12), *Rhizopus* species (n=4), and *Trichoderma* species (n=3). There was a significant association between hair length and dermatophytosis (p<0.0001). Associations between other fungal species and hair length are shown in Table-2.

**Table-2:** Fungal culture results of samples collected from 127 cats and the association between fungal species and hair length*.

Fungal species	Short-haired cats(n=64)	Long-haired cats(n=63)	Total(n=127)	Fisher’s exact test (p-value)
Dermatophyte				
*Microsporum* spp.	2	22	24	<0.0001
*Trichophyton* spp.	2	0	2	0.0959
Other fungal species				
*Aspergillus* spp.	12	31	43	0.0002
*Cladosporium* spp.	10	2	12	0.0125
*Rhizopus* spp.	2	2	4	0.9872
*Absidia* spp.	6	2	8	0.1416
*Penicillium* spp.	10	2	12	0.0125
*Nigrospora* spp.	2	0	2	0.0959
*Cladophialospora* spp.	1	1	2	0.9910
*Paecilomyces* spp.	2	1	3	0.5645
*Curvularia* spp.	2	3	5	0.6343
*Trichoderma* spp.	1	2	3	0.5462
*Chaetomium* spp.	2	0	2	0.0959
*Bipolaris* spp.	3	2	5	0.6600

* Statistical analysis was compared between short-haired and long-haired cats

There was significantly more positive Wood’s lamp test results seen in long-haired cats (10/63, 15.87%) compared with short-haired cats (3/64, 4.69%; p=0.0330) (Table-3). Images of the positive Wood’s lamp test results for short- and long-haired cats are shown in Figure-1. The prevalence of dermatophytosis in long-haired cats (22/63, 34.92% [95% CI, 22.94-46.90%]) was also significantly higher than that in short-haired cats (4/64, 6.25% [95% CI, 2.15-12.28%]; p<0.001) (Table-3). Moreover, there was a significant association between positive Wood’s lamp test and dermatophyte-positive culture (p=0.036; Table-3). The odds of long-haired cats having dermatophytosis were 8.05 (95% CI, 2.44-33.97) times greater than that in short-haired cats. It should be noted that the number of dermatophytosis-positive domestic short-haired (DSH) cats (2/50, 4.00%) was significantly lower than dermatophytosis-positive Persian (17/47, 36.17%; p<0.001) and long-haired mixed breed (3/7, 42.86%; p=0.011) cats (Table-3).

**Table-3:** Dermatophytosis diagnosed by Wood’s lamp test and dermatophyte cultures in cats categorized by hair coat length and cat breeds.

Cat groups	Wood’s lamp		Fisher’s exact-test (p-value)	Fungal culture dermatophytosis		Fisher’s exact-test (p-value)
	
	Positive	Negative		Positive	Negative	
Hair length*						
Short-haired cats (n=64)	3	61	-	4	60	-
Long-haired cats (n=63)	10	53	0.044	22	41	<0.001
Breed†						
DSH cats (n=50)	0	50	-	2	48	-
Scottish fold (n=5)	1	4	0.091	1	4	0.253
American/British short- haired cats (n=9)	2	7	0.021	1	8	0.397
Persian (n=47)	8	39	0.002	17	30	<0.001
Maine coon (n=9)	2	7	0.021	2	7	0.106
Mixed breed (hair length>3 cm; n=7)	0	7	0.275	3	4	0.011
Summary	13	14	-	26	101	0.036#

* Statistical analysis was compared with short-haired cats,

† statistical analyses were compared with DSH cats,

# statistical analysis was compared between Wood’s lamp test versus fungal culture

The overall sensitivity and specificity of the Wood’s lamp test for diagnosing *M. canis* infection were 37.5% (95% CI, 21.2-57.3%) and 96.1% (95% CI, 90.4-98.5%), respectively (Table-4). The positive and negative predictive values of the Wood’s lamp test for diagnosing *M. canis* infection were 69.2% (95% CI, 42.4-87.3%) and 86.8% (95% CI, 79.4-91.9%), respectively (Table-4).

**Table-4:** Sensitivity, specificity, positive predictive value, negative predictive value, and 95% CI of Wood’s lamp for detecting *Microsporum* species in short-haired cats, long-haired cats, and total sample.

Measurement	Total cats	Short-haired cats	Long-haired cats
		
	(n=127)	(n=64)	(n=63)
Sensitivity (%; 95% CI)	37.5 (21.2-57.3)	50 (9.5-90.5)	36.4 (24.3-47.2)
Specificity (%; 95% CI)	96.1 (90.4-98.5)	96.8 (89.0-99.1)	95.1 (83.9-98.7)
Positive predictive value (%; 95% CI)	69.2 (42.4-87.3)	33.3 (6.1-79.2)	80.0 (49.0-94.3)
Negative predictive value (%; 95% CI)	86.8 (79.4-91.9)	98.4 (91.3-99.7)	73.6 (60.4-83.6)

CI: Confidence interval

**Figure-4:**
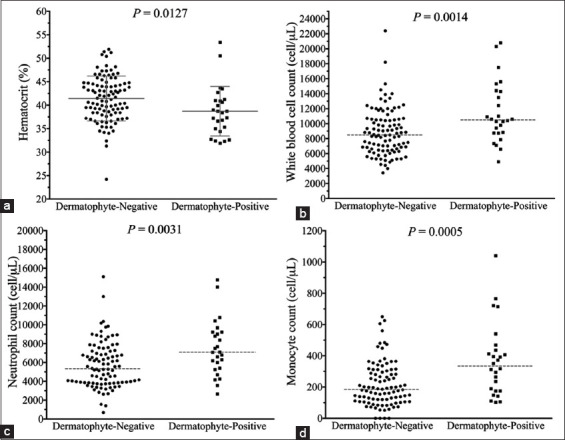
Scatter plots of hematology parameters of cats with negative and positive results for dermatophytosis. (a) Hematocrit, (b) white blood cell count, (c) neutrophil count, and (d) monocyte count. The dashed gray lines represent median. The solid gray lines represent mean and standard deviation.

The median hematocrit in cats with dermatophytosis was significantly lower than in cats without dermatophytosis (38.55% [31.9-53.4%] vs. 41.5% [24.2-51.9%], p=0.0127; Figure-4). The median (range) of white blood cell count, neutrophil count, and ­monocyte count in cats with dermatophytosis was significantly higher than those without dermatophytosis (white blood cell count: 10,500 (4900-20,800) cell/μL vs. 8480 (3420-22,400) cell/μL, p=0.0014; neutrophil count: 7096 (2646-14,768) cell/μL vs. 5338 (383-15,106) cell/μL, p=0.0031; monocyte count: 334 (103-1040) cell/μL vs. 184 (0-650) cell/μL, p=0.0005; (Figure-4). However, the median lymphocyte count and eosinophil count of cats with dermatophytosis did not differ significantly from those in cats without dermatophyte infection (lymphocyte count: 2173 [375-8064] cell/μL and 2575 [742-7105] cell/μL, p=0.1936; eosinophil count: 531 [197-2288] cell/μL vs. 481 [62-1885] cell/μL, p=0.0729).

**Figure-5:**
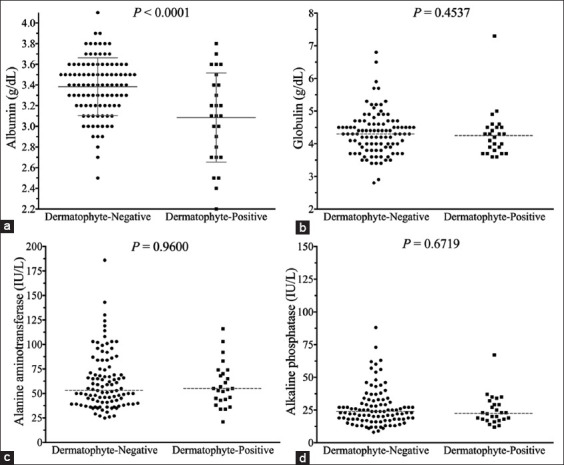
Scatter plots of blood chemistry parameters of cats with and without dermatophytosis. (a) Albumin, (b) globulin, (c) alanine aminotransferase, and (d) alkaline phosphatase. The dashed gray lines represent median. The solid gray lines represent mean and standard deviation.

The median total protein and albumin levels in cats with dermatophytosis were significantly lower than in cats without dermatophyte infection (total protein: 7.45 [6.2-9.5] g/dL vs. 7.7 [6.4-10.5] g/dL, p=0.0055; albumin: 3.1 [2.2-3.8] g/dL vs. 3.4 [2.5-4.1] g/dL, p<0.0001; Figure-5). However, the median globulin level in cats with dermatophytosis did not differ significantly from those in cats without dermatophyte infection (globulin: 4.3 [3.6-7.3] g/dL vs. 4.3 [2.8-6.8], p=0.4537; Figure-5). The median blood urea nitrogen and creatinine levels in cats with dermatophytosis were significantly lower than in cats without dermatophyte infection (blood urea nitrogen: 21.5 [13-36] mg/dL vs. 25 [16-39] mg/dL, p=0.0130; creatinine: 1.17 [0.71-1.72] mg/dL vs. 1.42 [0.96-2.63] mg/dL, p=0.0003); however, both blood urea nitrogen and creatinine levels were within the normal limits. The median alanine aminotransferase and alkaline phosphatase levels in cats with and without dermatophyte infection were within the normal limits and did not differ significantly (alanine aminotransferase: 55 [21-116] IU/L vs. 53 [25-186] and IU/L, p=0.9600; alkaline phosphatase: 22 [12-67] IU/L vs. 24 [8-88] IU/L, p=0.6719; Figure-5).

## Discussion

Dermatophytosis is a common cause of hair loss and scaling in cats, and dermatophytes can transmit to other species, including dogs and humans. In the present study, a survey of healthy short- and long-haired cats was conducted to detect subclinical infection with dermatophytes. Fungal cultures from 26 out of 127 cats (20.5%) were positive for dermatophytes, including *M. canis* (24/26, 92.3%) and *Trichophyton* species (2/26, 7.7%). The prevalence of dermatophyte contamination in long-haired cats (34.92%; 95% CI, 22.94-46.9%) was significantly higher than that in short-haired cats (6.25%; 95% CI, 2.15-12.28%), suggesting potential ringworm transmission to humans and animals that contact apparently healthy long-haired or Persian cats. Thus, dermatophyte examinations should be routinely performed due to the higher prevalence of dermatophytosis in long-haired and Persian cats.

Diagnosis of dermatophytosis in the present study was based on the results of fungal cultures using a combination of DTM, ESA, and SDA culture media. A previous study reported the prevalence of dermatophytosis in asymptomatic cats using DTM culture media for dermatophyte isolation [17]. The prevalence ranged from 2.1% to 15.7%, which was lower than the prevalence identified in the present study (20.5%) [17]. This discrepancy may due to the combined use of fungal agars, which increases the efficacy of dermatophyte detection. It may also be explained by the different geographic environments of the two studies.

The Wood’s lamp test, which uses a longwave ultraviolet light, is a useful screening tool for diagnosing dermatophyte infection on an animal skin [1]. Although the present study indicated a low sensitivity of Wood’s lamp for *M. canis* infection (37.5% of the infected cats), the test has a very high specificity of 96.1%. Another study reported the positive and negative predictive values of Wood’s lamp as 90% and 94%, respectively [19]. The overall positive predictive value was lower in our study. This may be due to a difference in the clinical dermatology status of the cats from the previous study, which involved testing cats that were clinically suspected of having dermatophyte infection.

*Aspergillus* species were the most abundant non-dermatophyte fungal species isolated in the present study. *Aspergillus* species were also previously identified as normal flora in healthy cats [16]. In the present study, *Aspergillus* species were identified in both short- and long-haired cats. The prevalence of *Aspergillus* infection in long-haired cats (49.2%; 95% CI, 36.38-62.11%) was significantly higher than that in short-haired cats (18.75%; 95% CI, 10.08-30.46%; p=0.0002). Our results indicated that the longer and thicker fur of long-haired cats harbored more fungi than that of short-haired cats.

Since the present survey was conducted in FeLV-negative and FIV-negative cats, the results may not reflect the actual prevalence of dermatophytosis in clinical settings. The prevalence of dermatophyte infections in cats infected with FeLV or FIV could be higher due to their compromised immunity and elevated risk of other opportunistic infections [18]. In the present study, Persian cats accounted for 74.6% of the long-haired cats, which may have skewed the data. Persian cats are known to have a higher susceptibility to dermatophyte fungi infections, and the influence of inheritance of the susceptible genes cannot be ruled out. In the present study, the number of positive dermatophytosis found in Persian cats (17/47, 36.17%) was significantly higher than that found in DSH cats (2/50, 4.00%). Our results were consistent with the findings of a previous study that report that identified dermatophytes in the hair coat of healthy Persian cats, especially from commercial catteries [20]. We also found that cats with dermatophytosis had significantly lower hematocrit and serum albumin levels compared with cats without dermatophytosis, suggesting that infected cats were in poorer health. Leukocytosis with elevated neutrophil and monocyte counts in cats with dermatophytosis may indicate stress or inflammatory response to fungal infections. Therefore, the hematology and blood chemistry profile of cats with dermatophytosis should be monitored.

## Conclusion

The present study revealed that long-haired cats were more susceptible to dermatophyte infection than short-haired cats. Long-haired cats are a potential source of dermatophyte pathogens due to their thicker coat, and fungal infections may spread to other cats and humans. Long-haired cats should be routinely evaluated for dermatophytosis using specific dermatophyte fungal media cultures as well as the Wood’s lamp test and blood profiles should be evaluated in cats with dermatophytosis.

## Authors’ Contributions

PS: Conceptualization, data curation, formal analysis, funding acquisition, software, writing – original draft. CB and PS: Investigation, methodology, validation, visualization. NT: Project administration and resources and supervision. PS and NT: Writing – original draft. PS, CB, and NT: Writing – review and editing. All authors read and approved the final manuscript.
